# Chinese Consumers’ Heterogeneous Preferences for the Front-of-Package Labeling on Fresh Pork: A Choice Experiment Approach

**DOI:** 10.3390/foods11182929

**Published:** 2022-09-19

**Authors:** Beixun Huang, Haijun Li, Zeying Huang, Jiazhang Huang, Junmao Sun

**Affiliations:** 1 Institute of Food and Nutrition Development, Ministry of Agriculture and Rural Affairs, Beijing 100081, China; 2 School of Information & Intelligence Engineering, University of Sanya, Sanya 572022, China; 3 Department of Logistic Service Center of Chinese Academy of Agricultural Sciences, Beijing 100081, China

**Keywords:** choice experiment, conditional logit model, fresh pork, front-of-package labeling, heterogeneity, preference

## Abstract

Excessive average daily pork intake of Chinese residents increases the risk of obesity and related chronic diseases. Understanding consumers’ preference for the Front-of-Package (FOP) labeling on fresh pork is of practical significance for designing an FOP labeling scheme that meets market demand and effectively guides moderate pork consumption. This study used the conditional logit model to reveal the stated preferences of 930 nationally representative respondents in China for FOP labeling attributes elicited by a choice experiment approach. The results indicated that respondents preferred the nutritional information to be printed in Chinese characters, the label size to be a quarter of the front package surface, the label color to be green, and the label price to account for 10% of the retail price of 500 g standard fresh pork. Moreover, these preferences were heterogeneous across the sample population due to respondents’ different levels of education and trust in labeling. People with primary and junior high school education preferred nutritional information in Chinese characters, while those with junior high education and above preferred green labeling. The higher the respondents’ trust in the labeling, the stronger their willingness to accept the appropriate FOP labeling price. Information campaigns and educational programs can be used to increase the acceptance of FOP labelling, particularly among consumers with low education levels and distrust of FOP labeling.

## 1. Introduction

Consuming more than the required daily portion sizes of meat leads to an unhealthy diet in China. The China Health and Nutrition Survey showed that the livestock and poultry meat intake of Chinese people aged between 18 and 59 years increased from 66.7 g/d in 1989 to 120 g/d in 2015, exceeding 60% of the maximum intake of 75 g/d recommended by The Balanced Diet Pagoda for Chinese Residents (2016) [1]. China is the world’s biggest pork consumer, with pork consumption accounting for 73.9% of the total meat intake [2]. Indeed, a high intake of red meat is associated with an increased risk of obesity and chronic diseases [3]. He et al. [4] found that red meat intake of more than 100 g per day is a risk factor for cardiovascular and metabolic diseases in the Chinese adult population.

Education and the popularization of science are the main interventions to reduce red meat intake in China [5]. In contrast, some developed countries such as Netherlands, Singapore, Sweden, and the United States, have implemented diversified interventions, including using the Front-of-Package (FOP) labeling for ratings, scoring, and health certifications for the nutritional value of fresh meat. The FOP nutrition labeling provides simplified information about the overall nutritional status or key nutritional components of food through symbols, graphics, text, or a combination thereof attached to the front of the food package [6]. It has been advocated by the World Health Organization (WHO) and the Codex Alimentarius Commission (CAC) [6,7]. It has been proved that using the FOP labeling on fresh agricultural products could help consumers quickly identify the nutritional status of food and increase healthy food purchasing [8,9]. Therefore, applying the FOP labeling to fresh pork, such as labeling that the raw pork is always kept at 0~4 °C during circulation and retail, is likely to promote Chinese residents’ healthy meat consumption.

Understanding consumers’ preferences for the labeling attributes is one of the key links in the scientific design of FOP labels, which has an important impact on improving the use rate of labels [10]. As Lancaster’s random utility theory noted, the total utility obtained by consumers from commodities purchases could be decomposed into the sum of utilities from various commodities’ attributes [11]. Information expression [10,12], size [12], color [10,13], and price [13] are vital attributes that affect consumers’ attention and understanding of FOP labels. Specifically, the FOP labeling of fresh pork is the aggregation of information expression, size, color, price, and other attributes. The total utility obtained by the consumers from the labeling is the sum of the utility from all labeling attributes, so rational consumers often choose the labeling scheme that could bring them the maximum utility.

At present, some studies have focused on consumers’ preference for nutrition labels for fresh agricultural products, including nutrition claims for aquatic products, unsaturated fatty acid omega-3 claims for eggs, and nutrition facts tables for pork [14,15,16]. The choice experiment is a method widely used in consumer preference surveys [15]; consumers’ preference for the food labeling attributes has shown population heterogeneity such as by gender, age, education level, personal annual income, attention to health products, cognition of food nutrition, and labeling trust [17,18,19,20].

The investigation method and heterogeneity analysis used by existing studies were worthy of reference. Most previous studies conducted preference surveys using a particular labeling as one of the food attributes rather than different attributes. Moreover, few studies focused on consumers’ preference for FOP labeling attributes of fresh agricultural products, providing insufficient guidance to improve nutrition labels’ content and format. This current study used a choice experiment design to evaluate the Chinese consumers’ heterogeneous preferences for the FOP labels on fresh pork.

## 2. Materials and Methods

### 2.1. Choice Experiment Design

The choice experiment is the design of choice sets composed of different product attributes as questionnaire alternatives under a virtual market environment for respondents to choose [21]. Important steps in the choice experiment are the design of choice sets and identifying the minimum sample size [22].

The choice set was based on the above relevant literature and characteristics of fresh pork on sales in China. As shown in Table 1, FOP labels contain four expressions of nutrition information: Chinese characters (e.g., low saturated fatty acid pork, high-protein pork), digits (e.g., 1~100 scores used for pork overall nutritional quality evaluation), letters (e.g., A to E used to indicate pork with high nutritional quality to low nutritional quality), and graphics (e.g., tick and keyhole used to show the pork with high nutritional quality). In general, the prominence of the FOP labeling seems directly related to the proportion of labeling size to the package front area. In order to examine the coordination relationship between the labeling size and the front package area, our study designed three labeling sizes, namely 6%, 13%, and 25% of a specific package (thereafter, the 6% labeling size, the 13% labeling size, and the 25% labeling size). The green and blue colors were used as two color attributes due to consumers’ familiarity and acceptance of colors from current food labels in China, including organic product logos, green food certifications, healthy choice logos, and health food labels. Regarding the approximate cost of FOP labels, our study designed three price attributes: 0%, 10%, and 15% of the retail price of standard fresh pork per 500 g (thereafter, the 0% labeling price, the 10% labeling price, and the 15% labeling price).

In this study, a total of 72 (4 × 3 × 2 × 3) attribute combinations were obtained by a full-factor design. It was not feasible for respondents to make choices within 2556 choice sets (72*71/2) if each choice set contained two different FOP labeling schemes. Eight representative choice sets (see the questionnaire in the supplementray material) were selected from 2556 choice sets by the orthogonal design method. Moreover, these choice sets were characterized by uniform dispersion, orderliness, and comparability. An example of the choice sets is shown in Table 2.

### 2.2. Sample Size Calculation

There were a variety of measurement methods for the minimum sample size in the choice experiment [23]. The method proposed by Orme and Johnson et al. is widely used, and the calculation formula is as follows [24,25].

(1) $$n=500\times \frac{L}{A\times C}\;\;\;\;\;\;\;\;\;$$

In Equation (1), *n* is the minimum sample size, *L* is the maximum attribute levels, *A* is the number of options in a choice set, and *C* is the number of choice sets. Calculations showed that this choice experiment required at least 84 samples.

### 2.3. Conditional Logit Model

In this study, the conditional logit model was used to analyze the effect of different FOP labeling attributes applied to fresh pork on consumers’ choice of labeling schemes. This model was developed by McFadden based on the binary logit model [26]. According to the discrete choice theory, consumers choose the labeling scheme based on utility maximization, so the random utility brought by individual i choosing the FOP labeling scheme j consisting of different attributes is expressed as follows [27]:

(2) $$U_{ij}=x_{ij}^{\prime }\beta +\varepsilon_{ij}$$

In Equation (2), $x_{ij}$ is a label attribute that varies with individual i (i=1,⋯,I) and scheme j (j=1,⋯,J). β  shows the effect of $x_{ij}\;$ on the random utility $U_{ij}$ but does not depend on the coefficients of scheme j.$\varepsilon_{ij}$ is the random error term.

Supposing that individual i believes that the utility brought by scheme j was higher than that of scheme k, the probability of individual i choosing scheme j can be written as follows:

(3) $$\begin{array}{c} P\left(y_{i}=j|\;x_{ij}\right)=P\left(U_{ij}\geq U_{ik}\;,\;\forall \;k\neq j\;\right)\\ =P\left(U_{ik}-U_{ij}\leq 0\;,\;\forall \;k\neq j\;\right)\\ =P\left(\varepsilon_{ik}-\varepsilon_{ij}\leq x_{ij}^{\prime }\beta -x_{ik}^{\prime }\beta \;,\;\forall \;k\neq j\;\right) \end{array}$$

In Equation (3), $y_{i}=j$ means that individual i chooses FOP labeling scheme j. The random utility brought by $U_{ij}$ and $U_{ik}$ for consumer i to choose labeling schemes j and k consists of different attributes. $\varepsilon_{ij}\;$and $\varepsilon_{ik}$ are random error terms. Assuming that {$\varepsilon_{ij}$} is an independently and identical distribution (IID), Equation (3) can be expressed as follows:

(4) $$P\left(y_{i}=j|\;x_{ij}\right)=\frac{e^{x_{ij}^{\prime }\beta }}{\sum_{k=1}^{J}e^{x_{ik}^{\prime }\beta }}$$

Equation (4) is the conditional logit model. The coefficient  β does not depend on the scheme, and there is no need to select the reference scheme and to normalize a part of β  to 0 [26].

Individual i’s choice of FOP labeling scheme j is $y_{ij}$, which can be represented by a dummy variable with 1 representing choice and 0 representing no choice, so the log-likelihood function of the conditional logit model is expressed as:

(5) $$y_{ij}=\ln\left[\frac{P\left(y_{i}=j|\;x_{ij}\right)}{1-P\left(y_{i}=j|\;x_{ij}\right)}\right]={\hat{\beta }}_{MLE}x_{ij}^{\prime }$$

In Equation (5), ${\hat{\beta }}_{MLE}$ is the estimated value of the regression coefficient obtained by maximum likelihood estimation.$\;x_{ij}$ refers to the explanatory variables such as the information expression of labeling, labeling size, labeling color, and labeling price whether individual i chooses label scheme j or not. 

In general, the coefficients of the nonlinear regression model can only be used to judge the direction of influence, and the magnitude of influence is usually expressed by odds ratio (OR). The odds ratio is exponential over the regression coefficients, namely $\mathrm{OR}=e^{{\hat{\beta }}_{MLE}}.$ If the odds ratio is greater than 1, an increase in x by one unit increases the probability of x occurring; otherwise, the probability decreases. In this study, the influence of different FOP labeling attributes on consumers’ labeling scheme selection behavior was explained by both regression coefficient and odds ratio.

### 2.4. Data Collection

Our proposed questionnaire (see Supplementary Material) included demographic information, cognition of fresh pork nutrition, trust in the FOP labeling, and the choice experiment for eliciting consumers’ preference for the FOP labeling. It was improved through the pre-survey of 40 adults in Beijing, China. For data availability, a paid online survey service was adopted from the Wenjuanxing (https://www.wjx.cn accessed on: 9 December 2021). Wenjuanxing, a well-known online survey company in China with a member database of 6.2 million registered members from 31 provinces, mainly provides paid data collection services for their clients. Besides at least 84 samples requested by Equation (1), this study determined the minimal number of representative random samples (*N* = 752) in China based on an allowable error of 3% and a confidence level of 90%, so we commissioned Wenjuanxing to collect 930 valid samples who were equally distributed between male and female. From December 2021 to January 2022, Wenjuanxing sent the questionnaire link to 33~37 adults randomly selected from each province/autonomous region/municipality in China to complete the survey online (i.e., 1106 samples in total), and about 84% responded. Before being investigated, all participants had been informed of the choice experiment instructions. Specifically, a virtual shopping scene of fresh pork along with two FOP labelings whose attributes levels were somewhat different was firstly described, then the respondents were asked to choose one from the two or not making a choice. Eight Chinese Yuan were offered to each respondent if their responses were careful and complete, but all respondents were not informed about the cash incentives before they participated in the survey. Finally, the data validity check produced 930 valid samples (i.e., 30 samples × 31 provinces), which were used for analysis.

## 3. Results 

### 3.1. Descriptive Statistics

As shown in Table 3, the distribution of respondents’ gender, ethnicity, education level, and residence was similar to that reported in Major Figures on the 2020 Population Census of China [23]. This validates a certain representativeness of the sample in the present study. 

Each of the respondents made selection decisions on sixteen labeling schemes from choice sets (*n* = 8), resulting in 14,880 (930 × 16) valid samples. Mathematical characteristics of relevant variables are shown in Table 4. About 46% of the respondents believed that different attributes of the FOP labeling applied to fresh pork are beneficial and chose the FOP labeling scheme designed in our study. Chinese characters, digits, letters, and graphics each accounted for 25% of the samples, and the three labeling sizes accounting for 6%, 13%, and 25% of the front area of the fresh pork package were 31.25%, 37.50%, and 31.25% of the whole samples, respectively. There was roughly a 50/50 split between green color and blue color. The three labeling prices accounting for 0%, 10%, and 15% of the average retail price of standard fresh pork per 500 g were 37.50%, 37.50%, and 25%, respectively. Regarding population characteristics, the respondents equally represented both sexes, with 44.60 years old on average and most having senior high school education. These families’ individual disposable income was about 48,113 Yuan on average. More than half of them often paid attention to the nutritional value of fresh pork and highly trusted the FOP labeling. However, respondents’ cognition of nutrition on fresh pork was not high. 

### 3.2. Full-Sample Regression Results

Stata statistical software 17.0 was used for the conditional logit regression analysis. The estimation results in Table 5 showed that four labeling attributes (information expression form, size, color, and price) all significantly affected respondents’ choice of FOP labeling schemes. As for the information expression forms of labels, the odds ratio of digits, letters, and graphics were 0.900, 0.781, and 0.899, respectively, which meant that the probability of choosing digits, letters, and graphics were 90%, 78.1%, and 89.9% of that of choosing Chinese characters, respectively. It further indicated that consumers would preferentially choose Chinese characters as the information expression form of the FOP labeling applied to fresh pork. The odds ratios of the FOP 13% and 25% labeling sizes were 1.103 and 1.164, respectively, indicating that the probabilities of consumers choosing these two labeling sizes were 1.103 and 1.164 times that of choosing the 6% labeling size. This finding reflected respondents’ conception of “the larger the labeling size, the better”. The odds ratio of choosing blue as the FOP labeling color was 0.659, meaning that the probability of consumers choosing blue was 65.9% of that of choosing green. This finding indicated that consumers preferred green FOP labeling to the blue one. As for the price of FOP labeling, the odds ratios of the 10% and 15% labeling price were 1.222 and 1.092, respectively, indicating that consumers were willing to pay for the FOP labeling of fresh pork, but they were more likely to accept the 10% labeling price.

### 3.3. Heterogeneity Analysis

The above empirical results indicated that each label attribute significantly impacted respondents’ choice of FOP labeling schemes, but how the impact varies across different populations needed further analysis. Firstly, demographic characteristics variables in this study were divided into continuous variables and categorical variables, and then t test and χ2 test were conducted on the four label attributes most preferred by respondents, including Chinese characters, the 25% labeling size, green color, and the 10% labeling price.

As shown in Table 6, the four label attributes did not significantly differ by respondents’ age and individual annual disposable income. The χ2 test results in Table 7 showed that only respondents’ education levels and trust degrees in FOP labeling passed the significant χ2 test in the four label attributes. Therefore, a regression analysis was used to measure the influence of labeling attributes on respondents’ choice of labeling schemes, based on sub-samples with different education levels and trust degrees in FOP labeling.

As shown in Table 8, only the labeling attribute of graphics expression had a statistically significant impact on the labeling choice behavior in the population with primary school education and below. 

The odds ratio of graphics expression was 0.193, indicating that the probability of choosing graphics as the expression form of FOP labeling information was only 19.3% of that of choosing Chinese characters. Compared with graphics, people with the lowest education level preferred Chinese characters. Letters as the information expression and blue as the labeling color affected the choice of the labeling scheme among the people with junior high school education level, and the odds ratio of the former and the latter were 0.637 and 0.565, respectively. It showed that the probability of choosing letters to express nutritional information was 63.7% of that of choosing Chinese characters, while the probability of choosing blue as the label color was only 56.5% of that of choosing green. It meant that when it came to letters and Chinese characters, respondents with a junior high school education level preferred Chinese characters, which was similar to those with primary school education or below; when it came to blue and green labels, they tended to prefer the green labeling. Color also significantly affected the labeling scheme of people with senior high school education. The odds ratio of blue labels was 0.719, indicating that the probability of choosing blue labeling was 71.9% of that of choosing green. All four labeling attributes significantly influenced respondents’ labeling choices in the undergraduate population. It indicated that college graduates paid attention to multiple attributes of the FOP labeling, and they favored the FOP labeling scheme printed in Chinese characters, with the 25% labeling size and green color, and having the 10% labeling price. Unlike people with college education, those with postgraduate education only valued the labeling color, while other labeling attributes had no significant effect. The odds ratio of blue was 0.650, indicating that people with graduate degrees or above also preferred the green color on the FOP labeling on fresh pork, as did people with middle school, high school, and college education.

As shown in Table 9, digit expressing information, graphics expressing information, and the 15% labeling price significantly affected the choice of labeling scheme for people who strongly distrusted the FOP labeling, and the odds ratios of the three attribute levels were 0.009, 0.010, and 0.015, respectively. Although this population did not trust the nutritional information conveyed by the FOP labeling, they preferred to convey the nutritional information of fresh pork with Chinese characters, as shown by the comparison between Chinese characters and digits and between Chinese characters and graphics. These residents were resistant to the FOP labeling due to distrust factors and wanted to implement the FOP labeling on fresh pork without charging any fees. As opposed to people who strongly distrusted the FOP labeling, those with trust in labeling were influenced by the labeling size and color. The odds ratio of the 25% labeling size and the blue color were 1.749 and 0.608, respectively, reflecting that this population did not believe the information conveyed by the FOP labeling to some extent. In terms of the labeling size and color, respondents who distrusted the FOP labeling preferred larger and green FOP labeling. The respondents with a low trust degree believed that information expression, labeling size, labeling color, and labeling price significantly influenced their choice of labeling scheme. Specifically, the odds ratios of letters as labeling information expression, the 25% labeling size, blue color, and the 10% labeling price were 0.784, 1.215, 0.732, and 1.179, respectively, which indicated that both respondents with the low trust level and those who strongly distrusted labeling preferred Chinese characters. Somewhat differently, people with trust in the FOP labeling occasionally were likely to accept the charged FOP labeling, while those respondents with less trust and average trust in the labeling were consistent in their preference for the larger and green labeling in terms of labeling size and color. All four labeling attributes had significant effects on the choice behavior of respondents with high trust in FOP labeling. These results indicated that these two populations preferred the FOP labeling printed in Chinese characters, with the 25% labeling size and green color, and having the 10% labeling price. Except for letters as the information expression form, blue color, and the 10% labeling price, other labeling attribute levels did not have a significant effect. The odds ratio showed that these respondents preferred FOP labeling expressed with Chinese characters and in green, and they were willing to pay the appropriate fee for the FOP labeling.

## 4. Discussion

The results of this study had two contributions. First, the choice experiment was used to accurately reveal the consumer’s preference for the FOP labeling on fresh pork, which provided theoretical support for the scientific formulation of the FOP labeling scheme on fresh pork in China. Second, the format and content of the FOP labeling favored by all respondents and the preference characteristics of different populations were identified through the regression analysis of the whole sample and the sub-samples, which provided enlightenment for the formulation and implementation of the FOP labeling intervention measures taken by other big red meat consumption countries. 

In this study, the preferred attributes of the FOP labeling for fresh pork were consistent with the findings of previous studies that the information expression form, labeling size, labeling color, and price of the labeling significantly attracted consumers’ attention [10,12,13]. Relevant studies focused on the FOP labeling applied to breakfast cereals and potato crisps [10,13] while our study took the labeling of fresh pork as an example, but consumers were all concerned about the information expression forms, labeling size, labeling colors, and price of the FOP labeling whether the labels were applied to pre-packaged food or fresh meat. In terms of information expression forms, Chinese consumers preferred the Chinese characters while Australian consumers preferred graphics mainly due to different interviewed residents and food [12]. As for the labeling color, Chinese consumers preferred green while consumers form U.K. preferred red and green [10]. In terms of the labeling price, Chinese consumers accepted the price that accounted for 10% of the retail price of fresh pork per 500 g, while British consumers could afford 0.3 GBP for the FOP labeling on potato chips. However, as for labeling size, consumers from China and Australia had preference for the labeling size that occupied a large area of the package. However, there were similarities in the larger labeling size preferred by both Chinese consumers and Australian consumers.

There existed heterogeneities in the preference for FOP labeling attributes of fresh pork among Chinese consumers, which was in line with the prior studies about heterogeneities of labeling preference in terms of education levels and trust degrees in the labeling [19,20]. However, previous studies only focused on labels. For example, Chinese consumers’ preference for pork import labels decreased with their higher education level [19], and Vietnamese consumers’ preference for VietGAP certification increased along with higher trust degree [20]. In contrast, consumers preferences’ heterogeneity regarding levels of labeling attributes was identified, and it was found that consumers who received different education levels had preferences for different information expression forms and colors, and consumers with different trust degrees in the labeling had different preferences for the paid labeling.

Several limitations were evident in this study. Firstly, the quality of online self-administered questionnaires taken may not be high. The online self-filled questionnaire is advantageous in terms of time- and labor-saving to collect data on many residents in a short time; however, it was probably difficult for respondents to understand the survey questions in the choice experiment due to the lack of investigators’ explanation. Secondly, the filling part of the choice experiment lacked the guidance of visual labeling graphics. The FOP labeling is an emerging label in China, but most residents have not understood it due to its low popularity rate and small propaganda intensity, especially in the application of fresh pork. Although the questionnaire survey in the present study introduced international FOP labels on fresh pork and their functions, the labeling scheme combining different attributes such as the information expression, labeling size, labeling color, and labeling price was not displayed through icons in the choice experiment. This weakened the respondents’ intuitive feeling of the FOP labeling and reduced the authenticity of stated preference to a certain extent. In future research, respondents’ understanding of the survey questions and added auxiliary decision-making materials such as pictures should be considered in the choice experiment to investigate consumers’ preference for FOP labeling attributes. Third, cross-term analysis of each labeling attribute was lacking in the conditional logit model. It was inferred based on findings of Ubilava et al. [28] that information expression, labeling size, labeling color, and labeling price on FOP labels existed simultaneously. However, there were perhaps substitutions (negative cross-term coefficients) or complementarity (positive cross-term coefficients) relationships between them, which were of great significance to promoting the organic combination of the FOP labeling attributes and improving consumers’ attention and utilization rates. However, labeling attributes were only listed as explanatory variables in the conditional logit model, and cross terms between attributes were not added in this study, which may be the future research direction.

## 5. Conclusions and Recommendation

The current study was carried out to determine Chinese adults’ preferences for different FOP labeling attributes of fresh pork. The findings revealed that respondents preferred the FOP labeling in Chinese characters, the labeling size to be a quarter of the front package surface, the labeling color to be green, and the labeling price to account for 10% of 500 g of standard fresh pork retail price. People with primary school, junior high school, and college educations liked Chinese characters better, while those with junior high education and above preferred green labeling to blue one. The residents with higher trust in the FOP labeling would rather pay the price for the labeling. The following policy recommendations are offered. First, the FOP labeling should be promoted to describe fresh pork’s nutritional characteristics or overall nutritional quality of fresh pork concisely and objectively by using Chinese characters, similar to those used in domestic nutrition claims and the healthy choice labeling. Second, the design of FOP labeling on fresh pork with green color and reasonable size should be promoted, but the color of the FOP labels should be different from the color used for labels on organic products and green foods. Meanwhile, the layout of various food labels on the front package of fresh pork should be coordinated, and the size of new FOP labels and existing labels should be harmonized and unified. Third, various forms of FOP labeling information dissemination should be implemented to improve the cognition level of consumers with low education and distrust in FOP labeling. 

## Figures and Tables

**Table 1:** Attributes and levels set in the choice.

Attribute	Attribute Levels
Labeling information expression	(1) Chinese Character (2) Digit (3) Letter (4) Graphic
Labeling size	(1) 6% of the front area of the fresh pork package (2) 13% of the front area of the fresh pork package (3) 25% of the front area of the fresh pork package
Labeling color	(1) Green (2) Blue
Labeling price	(1) 0 RMB (2) 10% of the average retail price of standard fresh pork per 500 g (3) 15% of the average retail price of standard fresh pork per 500 g

Note: the full meaning of RMB is Ren Min Bi.

**Table 2:** An example of a choice set.

Box 1	Option A	Option B	Option C
Labeling information expression	Digit	Chinese Character	Neither
Labeling size	6% of the front area of the package	25% of the front area of the package	
Labeling color	Green	Blue	
Labeling price	0 RMB	10% of the price of pork per 500 g	
I would choose: (Please mark only one box)	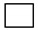	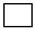	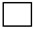

**Table 3:** Respondents’ individual characteristics.

Characteristics	Items	Samples	Percentage (%)	The 2020 Population Census Data (%)
Gender	Male	465	50	51.2
	Female	465	50	48.8
Age	0~14 years old	0	0	17.9
	15~59 years old	757	81.4	63.4
	60~65 years old	41	4.4	5.2
	more than 65 years old	132	14.2	13.5
Ethnic group	Han Ethnic Group	879	94.5	91.1
	Ethnic Minorities	51	5.4	8.9
Education level ^a^	Primary school or below	153	16.5	16.5
	Junior school	149	16.0	16.1
	Senior school	344	37.0	36.9
	Junior college or above	284	30.5	30.5
Residence	Urban area	558	60	63.9
	Rural area	372	40	36.1

Note: ^a^ the education levels in Table 3 were divided into four categories according to the classification of the national census data while junior college or above provided hereinbelow divided into both junior college or undergraduate and postgraduate or above were described.

**Table 4:** Variables measurement and descriptive statistics.

**Variable**	**Definition**	**Mean**	**Standard** **Deviation**	**Min.**	**Max.**	**Obs.**
*Dependent*						
Choice	No = 0; Yes = 1	0.46	0.50	0	1	14,880
*Independent*						
Labeling information expression	Chinese character Digit Letter Graphic	— — — —	— — — —	— — — —	— — — —	3720 3720 3720 3720
Labeling size	6% of the front area of the package 13% of the front area of the package 25% of the front area of the package	— — —	— — —	— — —	— — —	4650 5580 4650
Labeling color	Green Blue	— —	— —	— —	— —	7440 7440
Labeling price	0 RMB 10% of the price of pork per 500 g 15% of the price of pork per 500 g	— — —	— — —	— — —	— — —	5580 5580 3720
Gender	Female = 0; Male = 1	0.50	0.50	0	1	14,880
Age	Years	44.60	10.43	18	73	7440
Education level	Primary school or below	—	—	—	—	2448
	Junior school	—	—	—	—	2384
	Senior school	—	—	—	—	5504
	Junior college or undergraduate	—	—	—	—	3120
	Postgraduate or above	—	—	—	—	1424
Individual annual disposable income	Chinese Yuan	48,113.63	61,822.35	900	950,000	14,880
Often pay attention to the nutritional value of fresh pork	No = 0; Yes = 1	0.53	0.50	0	1	14880
Cognition of fresh pork nutrition ^a^	Know not at all Know not much Know a little Know somewhat well, Know quite well Know very well	— — — — — —	— — — — — —	— — — — — —	— — — — — —	704 2848 5664 4240 1216 208
Trust in the FOP labeling	Not at all	—	—	—	—	160
	Rarely	—	—	—	—	400
	Occasionally	—	—	—	—	2560
	Mostly	—	—	—	—	7312
	Very much	—	—	—	—	4480

Source: Authors’ own calculation. One US dollar was equal to 6.338 Chinese Yuan, and One Euro was equal to 7.121 Chinese Yuan from December 2021 to January 2022. ^a^ Each respondent’s cognition of fresh pork nutrition level was evaluated by five declarative questions. Whether respondents knew about healthy diets or not was evaluated by five questions from The Chinese Dietary Guidelines [5] that were included in the online questionnaire (see Supplementary Materials for details). Zero, one, two, three, four, and five correct answers indicated know not at all, know not much, know a little, know somewhat well, know quite well, know very well, respectively.

**Table 5:** Estimation results of conditional logit model for the total sample.

Independent Variables	Coefficients	Odds Ratio
Labeling information expression (Chinese character is the reference group)		
Digit	−0.106 ** (0.049)	0.900 ** (0.044)
Letter	−0.247 *** (0.049)	0.781 *** (0.038)
Graphic	−0.106 ** (0.052)	0.899 ** (0.047)
Labeling size (6% of the front area of the package is the reference group)		
13% of the front area of the package	0.098 ** (0.042)	1.103 ** (0.047)
25% of the front area of the package	0.152 *** (0.043)	1.164 *** (0.050)
Labeling color (green is the reference group)		
Blue	−0.417 *** (0.034)	0.659 *** (0.023)
Labeling price (0 RMB is the reference group)		
10% of the price of pork per 500 g	0.201 *** (0.039)	1.222 *** (0.048)
15% of the price of pork per 500 g	0.088 * (0.046)	1.092 * (0.050)
Log likelihood	−8213.95	
LR χ2(8)	215.17 ***	
Observations	14288	

Note: standard errors in parentheses; * *p* < 0.1, ** *p* < 0.05, *** *p* < 0.01.

**Table 6:** Preference for labels’ attributes by consumers with different characteristics (continuous variables).

Attributes	Mean	
	Age	Individual Annual Disposable Income
Preference for labels with Chinese character		
Yes	35.329	47,599.580
No	35.852	48,598.140
t statistic	1.529	0.492
Preference for labels whose size is 25% of the front area of the package		
Yes	35.173	48,040.570
No	36.005	48,183.540
t statistic	2.719 ***	0.079
Preference for labels whose color is green		
Yes	35.488	48,262.360
No	35.714	47,957.180
t statistic	0.934	−0.213
Preference for labels whose price is 10% of the price of pork		
Yes	35.205	48,580.440
No	35.969	47,672.200
t statistic	2.736 ***	−0.548

Note: *** *p* < 0.01.

**Table 7:** Preference for labels’ attributes by consumers with different characteristics (categorical variables).

Personal Characteristics	Numbers of Respondents Preferring			
	Preference for Labels with Chinese Character	Preference for Labels Whose Size Is 25% of the Front Area of the Package	Preference for Labels Whose Color Is Green	Preference for Labels Whose Price Is 10% of the Price of Pork
Male	892	1142	1847	1330
Female	913	1132	1967	1382
χ2 statistic	0.475	0.086	7.747 ***	1.940
Primary school or below	617	696	852	540
Junior school	420	467	851	594
Senior school	936	1098	1914	1355
Junior college or undergraduate	516	638	1008	741
Postgraduate or above	304	358	562	413
χ2 statistic	8.047 *	23.327 ***	20.665 ***	21.509 ***
Often pay attention to the nutritional value of pork	965	1093	2108	1470
Not often pay attention to the nutritional value of pork	840	1181	1706	1242
χ2 statistic	0.626	1.323	19.225 ***	4.196 **
Not know the pork nutrition at all	79	96	172	120
Not know the pork nutrition well	327	378	711	475
Know the pork nutrition a little	699	900	1470	1055
Know the pork nutrition somewhat well	521	678	1062	787
Know the pork nutrition quite well	151	184	350	236
Know the pork nutrition very well	28	38	49	39
χ2 statistic	4.172	24.988 ***	13.836 **	11.682 **
Not trust in the FOP labeling at all	11	17	15	14
Trust in the FOP labeling rarely	38	53	81	62
Trust in the FOP labeling occasionally	280	358	580	415
Trust in the FOP labeling mostly	924	1157	1921	1352
Trust in the FOP labeling very much	552	689	1217	869
χ2 statistic	20.902 ***	15.070 ***	74.309 ***	38.310 ***

Note: * *p* < 0.1, ** *p* < 0.05, *** *p* < 0.01.

**Table 8:** Respondents with different education levels as determined by the conditional logit regression.

Independent Variables	Respondents with Primary School or below Level		Respondents with Junior School Level		Respondents with Senior School Level		Respondents with Junior College or Undergraduate Level		Respondents with Postgraduate or above Level	
	Coefficients	Odds Ratio	Coefficients	Odds Ratio	Coefficients	Odds Ratio	Coefficients	Odds Ratio	Coefficients	Odds Ratio
Labeling information expression (Chinese character is the reference group)										
Digit	−1.440 (0.938)	0.237 (0.222)	0.339 (0.269)	1.403 (0.377)	0.068 (0.169)	1.071 (0.181)	−0.123 ** (0.055)	0.884 ** (0.048)	−0.285 (0.195)	0.752 (0.147)
Letter	−1.070 (0.924)	0.343 (0.317)	−0.452 * (0.272)	0.637 * (0.173)	−0.212 (0.169)	0.809 (0.137)	−0.246 *** (0.055)	0.782 *** (0.043)	−0.161 (0.195)	0.851 (0.166)
Graphic	−1.644 * (0.987)	0.193 * (0.191)	0.197 (0.280)	1.218 (0.342)	0.157 (0.178)	1.170 (0.208)	−0.149 ** (0.057)	0.862 ** (0.050)	−0.001 (0.206)	0.999 (0.205)
Labeling size (6% of the front area of the package is the reference group)										
13% of the front area of the package	0.124 (0.794)	1.132 (0.899)	0.117 (0.231)	1.125 (0.260)	0.087 (0.146)	1.091 (0.159)	0.098 ** (0.047)	1.103 ** (0.052)	0.108 (0.168)	1.114 (0.187)
25% of the front area of the package	0.892 (0.830)	2.439 (2.025)	−0.319 (0.236)	0.727 (0.172)	−0.125 (0.147)	0.882 (0.130)	0.198 *** (0.047)	1.219 *** (0.058)	0.151 (0.169)	1.163 (0.197)
Labeling color (green is the reference group)										
Blue	0.389 (0.645)	1.476 (0.952)	−0.571 *** (0.187)	0.565 *** (0.106)	−0.329 *** (0.118)	0.719 *** (0.085)	−0.423 *** (0.038)	0.655 *** (0.025)	−0.430 *** (0.135)	0.650 *** (0.088)
Labeling price (0 RMB is the reference group)										
10% of the price of pork per 500 g	−0.548 (0.736)	0.578 (0.426)	0.151 (0.212)	1.163 (0.247)	0.038 (0.134)	1.039 (0.139)	0.231 *** (0.043)	1.260 *** (0.055)	0.092 (0.154)	1.096 (0.169)
15% of the price of pork per 500 g	0.519 (0.884)	0.570 (0.041)	−0.120 (0.252)	0.887 (0.224)	−0.108 (0.158)	0.897 (0.142)	0.119 ** (0.051)	1.127 ** (0.058)	−0.005 (0.183)	0.995 (0.182)
Log likelihood	−23.930		279.752		−697.676		−6670.152		−523.911	
LR χ2(8)	7.97		20.27 ***		13.92 *		195.26 ***		14.81 *	
Observations	2448		2384		5504		3120		1424	

Note: standard errors in parentheses; * *p* < 0.1, ** *p* < 0.05, *** *p* < 0.01.

**Table 9:** Respondents with different trust degrees in the FOP labeling as determined by the conditional logit regression.

Independent Variables	Respondents with No Trust in the FOP Labeling at All		Respondents with Trust in the FOP Labeling Rarely		Respondents with Trust in the FOP Labeling Occasionally		Respondents with Trust in the FOP Labeling Mostly		Respondents with Trust in the FOP Labeling Very Much	
	Coefficients	Odds Ratio	Coefficients	Odds Ratio	Coefficients	Odds Ratio	Coefficients	Odds Ratio	Coefficients	Odds Ratio
Labeling information expression (Chinese character is the reference group)										
Digit	−22.798 *** (0.829)	0.009 *** (0.001)	−0.419 (0.341)	0.658 (0.224)	−0.102 (0.124)	0.903 (0.112)	0.170 ** (0.069)	0.844 ** (0.059)	0.034 (0.089)	1.034 (0.092)
Letter	−0.921 (1.076)	0.398 (0.428)	0.003 (0.335)	1.003 (0.336)	−0.243 ** (0.124)	0.784 ** (0.097)	−0.312 *** (0.069)	0.732 *** (0.051)	−0.152 * (0.089)	0.859 * (0.076)
Graphic	−23.636 *** (1.206)	0.010 *** (0.001)	−0.266 (0.355)	0.767 (0.272)	−0.186 (0.130)	0.830 (0.108)	−0.172 ** (0.073)	0.842 ** (0.061)	0.087 (0.093)	1.091 (0.102)
Labeling size (6% of the front area of the package is the reference group)										
13%	0.047 (0.850)	1.048 (0.891)	0.049 (0.292)	1.050 (0.306)	0.093 (0.107)	1.098 (0.117)	0.108 * (0.060)	1.114 * (0.067)	0.093 (0.076)	1.097 (0.084)
25%	23.337 (0.001)	0.001 (0.001)	0.559 * (0.293)	1.749 * (0.513)	0.195 * (0.107)	1.215 * (0.130)	0.188 *** (0.060)	1.207 *** (0.073)	0.017 (0.077)	1.017 (0.078)
Labeling color (green is the reference group)										
Blue	0.922 (0.840)	2.513 (2.110)	−0.498 ** (0.235)	0.608** (0.143)	−0.313 *** (0.086)	0.732*** (0.063)	−0.401 *** (0.048)	0.670*** (0.032)	−0.512 *** (0.062)	0.600 *** (0.037)
Labeling price (0 RMB is the reference group)										
10% of the price of pork per 500 g	0.924 (0.985)	2.519 (2.481)	0.462 (0.267)	0.587 (0.424)	0.164 * (0.098)	1.179 * (0.115)	0.175 *** (0.055)	1.192 *** (0.066)	0.235 *** (0.070)	1.265 *** (0.089)
15% of the price of pork per 500 g	23.310 *** (1.185)	0.015 *** (0.001)	0.145 (0.323)	1.156 (0.374)	0.164 (0.116)	1.178 (0.136)	0.157 ** (0.065)	1.170 ** (0.076)	−0.097 (0.083)	0.908 (0.075)
Log likelihood	−28.046		−175.288		−1305.438		−4139.854		−2534.561	
LR χ2(8)	33.67 ***		16.53 **		23.76 ***		109.12 ***		93.62 ***	
Observations	80		336		2288		7168		4416	

Note: standard errors in parentheses; * *p* < 0.1, ** *p* < 0.05, *** *p* < 0.01.

## Data Availability

Data is contained within the article.

## Supplementary Materials

The following supporting information can be downloaded at: https://www.mdpi.com/article/10.3390/foods11182929/s1, Supplementary Materials: The questionnaire.
