# Supplementary material for: Chinese Consumers’ Heterogeneous Preferences for the Front-of-Package Labeling on Fresh Pork: A Choice Experiment Approach
Source: Foods. 2022 Sep 19;11(18):2929. doi: 10.3390/foods11182929 (PMC9498404; doi:10.3390/foods11182929)
Supplement: Supplementary file 1 [file foods-11-02929-s001.zip › foods-1892931-supplementary.pdf]

The questionnaire

Section 1 Demographic characteristics

Q1-1 Your gender: A. Male B. Female

Q1-2 Your age: ______years old

Q1-3 Are you Han Chinese people? A. Yes B. No

Q1-4 Your residence: A. Urban area B. Rural area

Q1-5 Your education level: A. Primary school or below B. Junior school C. Senior school D. Junior college or undergraduate E. Postgraduate or above

Q1-6 Your annual disposable income: ______Chinese Yuan

Q1-7 There are: ______ permanent residents in your family

Section 2 Test questions on cognition of fresh pork nutrition

Q2-1 What is the minimum fat content of pork that can be called fat pork?

A.10% B.20% C.30% D.40% E.50%

Q2-2 What is the maximum fat content of pork that can be called lean pork?

A.10% B.20% C.30% D.40% E.50%

Q2-3 Which of the following parts of pork has the lowest fat content?

A. Pork neck B. Griskin C. Streaky pork D. Elbow meat E. Pork rump

Q2-4 What percentage of saturated fatty acids is in pork fat?

A.25%-35% B. 35%-45% C.45%-55% D. 55%-65% E. 65%-75%

Q2-5 What is the maximum amount of pork we can eat in a day for a healthy diet?

A. 25 g B.50 g C.75 g D.100 g E.125 g

Section 3 Trust in the FOP labeling applied to fresh pork

There is a nutrition labeling that uses a symbol or graphic to show the nutritional status of fresh pork, which is known as front of package (FOP) nutrition labeling. The United States, the Netherlands, Sweden, Singapore have applied FOP nutrition labeling to fresh pork. Consumers could know the nutritional value of fresh pork through the symbol, 0~3 stars and 1~100 scores shown in Table S1.

Table S1. The FOP labelling applied to fresh pork internationally

FOP nutrition labeling (one example)	Swedish Keyhole symbol	American Heart-check mark	Singapore Healthier choice symbol	Choices logo in the Dutch	American Guiding stars labeling	American NuVal labeling
Role	Low saturated fatty acid, low sodium fresh pork labelled with the above keyhole	High overall nutritional quality of fresh pork lablled with the above red heart	Low saturated fatty acid, low sodium fresh pork labelled with the above pyramid	High overall nutritional quality of fresh pork lablled with the above tick graphic	Overall nutritional quality of fresh pork lablled with 0~3 stars. The more stars, the higher the	Overall nutritional quality of fresh pork lablled with 1~100 scores. The more scores, the

	graphic	graphic	graphic	overall nutritional quality	higher the overall nutritional quality

Q3-1 Do you often pay attention to the nutritional value of fresh pork?
A. Yes B. No

Q3-2 If the FOP nutrition labeling similar to Table S1 was applied to fresh pork in China, would you trust the labeling information?
A. very much
B. mostly
C. occasionally
D. rarely
E. not at all

Section 4 The choice experiment for eliciting consumers' preference for the FOP labeling applied to fresh pork

Suppose that when you visit the fresh pork case in your supermarket during a given month you are presented with two choices of the front of package (FOP) labels on fresh pork (Option A and Option B). Label A and Label B have different attributes but we have described below for you. While many attributes vary from Label A to Label B. The following are descriptions of the attributes but may vary from Label A to Label B:

Labeling information expression. The FOP labeling shows nutritional information of fresh pork by one way.

Labeling size. The size of FOP labeling is expressed in percentages of the front area of the fresh pork package.

Labeling color. The FOP labeling is expressed a single color.

Labeling price. The price of FOP labeling is expressed in percentages of the average retail price of common fresh pork per 500 grams.

Consider each of the following eight boxes below as separate sets of choices. In each of the eight boxes please select the FOP labeling choice (Option A, Option B or Option C) that best matches your preferences:

Box 1	Option A	Option B	Option C
Labeling information expression	Digit	Character	Neither
Labeling size	6% of the front area of the package	25% of the front area of the package	
Labeling color	Green	Blue	
Labeling price	0 RMB	10% of the price of pork per 500 grams	
I would choose:(Please mark only one box)	☐	☐	☐

Box 2	Option A	Option B	Option C
Labeling information expression	Character	Digit	Neither
Labeling size	6% of the front area of the package	25% of the front area of the package	
Labeling color	Blue	Green	
Labeling price	0 RMB	10% of the price of pork per 500 grams	
I would choose:(Please mark only one box)	☐	☐	☐

Box 3	Option A	Option B	Option C
Labeling information expression	Letter	Digit	Neither
Labeling size	25% of the front area of the package	13% of the front area of the package	
Labeling color	Green	Blue	
Labeling price	0 RMB	15% of the price of pork per 500 grams	
I would choose:(Please mark only one box)	☐	☐	☐

Box 4	Option A	Option B	Option C
Labeling information expression	Character	Digit	Neither
Labeling size	13% of the front area of the package	6% of the front area of the package	
Labeling color	Green	Blue	
Labeling price	0 RMB	10% of the price of pork per 500 grams	
I would choose:(Please mark only one box)	☐	☐	☐

Box 5	Option A	Option B	Option C
Labeling information expression	Graphic	Letter	Neither
Labeling size	13% of the front area of	25% of the front area of	

	the package	the package	
Labeling color	Blue	Green	
Labeling price	10% of the price of pork per 500 grams	15% of the price of pork per 500 grams	
I would choose:(Please mark only one box)	☐	☐	☐

Box 6	Option A	Option B	Option C
Labeling information expression	Graphic	Character	Neither
Labeling size	13% of the front area of the package	6% of the front area of the package	
Labeling color	Blue	Green	
Labeling price	15% of the price of pork per 500 grams	10% of the price of pork per 500 grams	
I would choose:(Please mark only one box)	☐	☐	☐

Box 7	Option A	Option B	Option C
Labeling information expression	Letter	Digit	Neither
Labeling size	13% of the front area of the package	25% of the front area of the package	
Labeling color	Green	Blue	
Labeling price	10% of the price of pork per 500 grams	0 RMB	
I would choose:(Please mark only one box)	☐	☐	☐

Box 8	Option A	Option B	Option C
Labeling information expression	Graphic	Letter	Neither
Labeling size	6% of the front area of the package	13% of the front area of the package	
Labeling color	Green	Blue	
Labeling price	15% of the price of pork per 500 grams	0 RMB	
I would choose:(Please mark only one box)	☐	☐	☐
